# Supplementary material for: Data valuation for medical imaging using Shapley value and application to a large-scale chest X-ray dataset
Source: Sci Rep. 2021 Apr 16;11:8366. doi: 10.1038/s41598-021-87762-2 (PMC8052417; doi:10.1038/s41598-021-87762-2)
Supplement: Supplementary file 2 — Supplementary Data 2. [file 41598_2021_87762_MOESM2_ESM.docx]

**Legend for Supplementary Data**

**Supplementary Data. We provide the radiologists’ labels of the 300 chest X-rays (i.e. 100 most valuable, 100 least valuable and 100 randomly sampled images from the training set) and their TMC-Shapley values.** Column “Image Index” shows the image file names as they appear in ChestX-ray14 dataset. Column “Pneumonia (ChestX-ray14)” shows the original pneumonia labels (1 for pneumonia and 0 for no pneumonia) in ChestX-ray14. Columns “Pneumonia (Radiologist 1)”, “Pneumonia (Radiologist 2)” and “Pneumonia (Radiologist 3)” show the radiologists’ labels. Column “TMC-Shapley Value” shows the TMC-Shapley values.
