# Supplementary material for: Data valuation for medical imaging using Shapley value and application to a large-scale chest X-ray dataset
Source: Sci Rep. 2021 Apr 16;11:8366. doi: 10.1038/s41598-021-87762-2 (PMC8052417; doi:10.1038/s41598-021-87762-2)
Supplement: Supplementary file 3 — Supplementary Files. [file 41598_2021_87762_MOESM3_ESM.docx]

**Data Valuation for Medical Imaging Using Shapley Value and Application to a Large-scale Chest X-ray Dataset**

**Supplementary Information**

Siyi Tang, Amirata Ghorbani, Rikiya Yamashita, Sameer Rehman, Jared A. Dunnmon, James Zou, Daniel L. Rubin^*^

**^*^Address correspondence to:**

Daniel L. Rubin

Professor of Biomedical Data Science and Radiology

Stanford University

Email: dlrubin@stanford.edu

**Supplementary Table S1.** Pearson’s correlation coefficients between (a) TMC-Shapley values and (b) G-Shapley values approximated using features from different pre-trained convolutional neural networks, different supervised learning algorithms and different validation and held-out test sets.

Abbreviations: LR: logistic regression; MLP: multi-layer perceptron.

1. **Pearson’s correlation coefficients for TMC-Shapley values**

|  | **CheXNet features, LR (Original)** | **ResNet-50 features, LR** | **CheXNet features, MLP** | **New validation & held-out test sets** |
| --- | --- | --- | --- | --- |
| **CheXNet features, LR (Original)** | - | - | - | - |
| **ResNet-50 features, LR** | 0.851 | - | - | - |
| **CheXNet features, MLP** | 0.926 | 0.814 | - | - |
| **New validation & held-out test sets** | 0.980 | 0.843 | 0.945 | - |

1. **Pearson’s correlation coefficients for G-Shapley values**

|  | **CheXNet features, LR (Original)** | **ResNet-50 features, LR** | **CheXNet features, MLP** | **New validation & held-out test sets** |
| --- | --- | --- | --- | --- |
| **CheXNet features, LR (Original)** | - | - | - | - |
| **ResNet-50 features, LR** | 0.624 | - | - | - |
| **CheXNet features, MLP** | 0.928 | 0.652 | - | - |
| **New validation & held-out test sets** | 0.960 | 0.624 | 0.973 | - |

**Supplementary Table S2.** Inter-reader agreement for (a) 100 least valuable images, (b) 100 most valuable images and (c) 100 randomly sampled images.

1. **100 Least valuable images**

|  | **Pneumonia** | **No Pneumonia** | **Unsure** |
| --- | --- | --- | --- |
| **# Agreed by Three Radiologists** | 10 | 18 | 0 |
| **# Agreed by Two Radiologists** | 42 | 14 | 6 |
| **# Disagreed by Three Radiologists** | 10 | | |

1. **100 Most valuable images**

|  | **Pneumonia** | **No Pneumonia** | **Unsure** |
| --- | --- | --- | --- |
| **# Agreed by Three Radiologists** | 24 | 7 | 0 |
| **# Agreed by Two Radiologists** | 41 | 15 | 7 |
| **# Disagreed by Three Radiologists** | 6 | | |

1. **100 Randomly sampled images**

|  | **Pneumonia** | **No Pneumonia** | **Unsure** |
| --- | --- | --- | --- |
| **# Agreed by Three Radiologists** | 3 | 64 | 0 |
| **# Agreed by Two Radiologists** | 13 | 15 | 1 |
| **# Disagreed by Three Radiologists** | 4 | | |


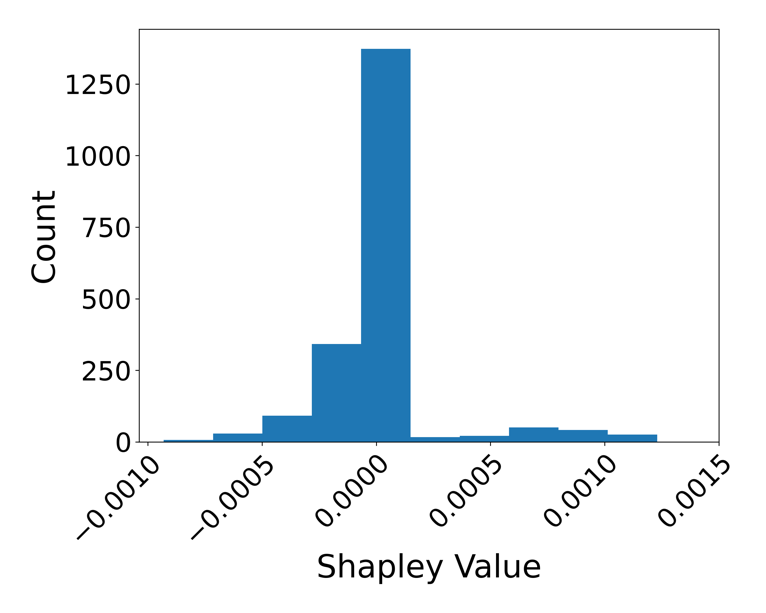


**Supplementary Figure S1. Histogram of TMC-Shapley values for training data.** Most data points have Shapley values around 0. Data points with negative Shapley values (41.5%) on average harm the performance of the pneumonia detection algorithm, whereas data points with high Shapley values are valuable for pneumonia detection.

**
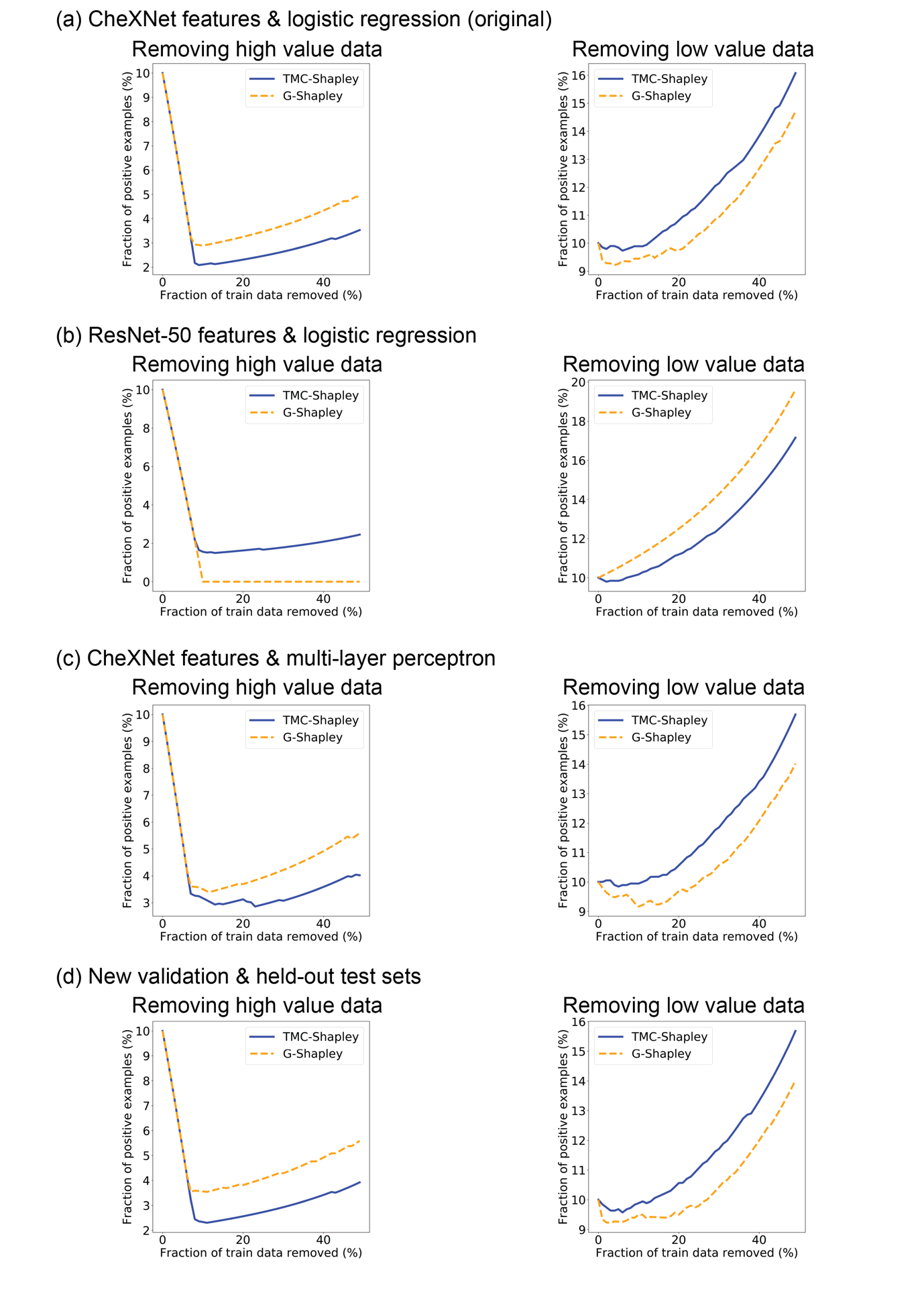
**

**Supplementary Figure S2. Fraction of positive training examples when training data were removed starting from the most (least) valuable data point.** (a) Input features were extracted from a pre-trained CheXNet^1^ and logistic regression was used as the learning algorithm. (b) Input features were extracted from a pre-trained ResNet-50^2^ and logistic regression was used as the learning algorithm. (c) Input features were extracted from a pre-trained CheXNet and multi-layer perceptron was used as the learning algorithm. (d) We sampled different validation and held-out sets. Pneumonia prediction accuracy was used as the performance metric for all the experiments. As training data were removed starting from the most valuable data points, the fraction of positives quickly dropped from 10% to 2%, suggesting that most positive training examples were identified as valuable by the Shapley algorithms.

**
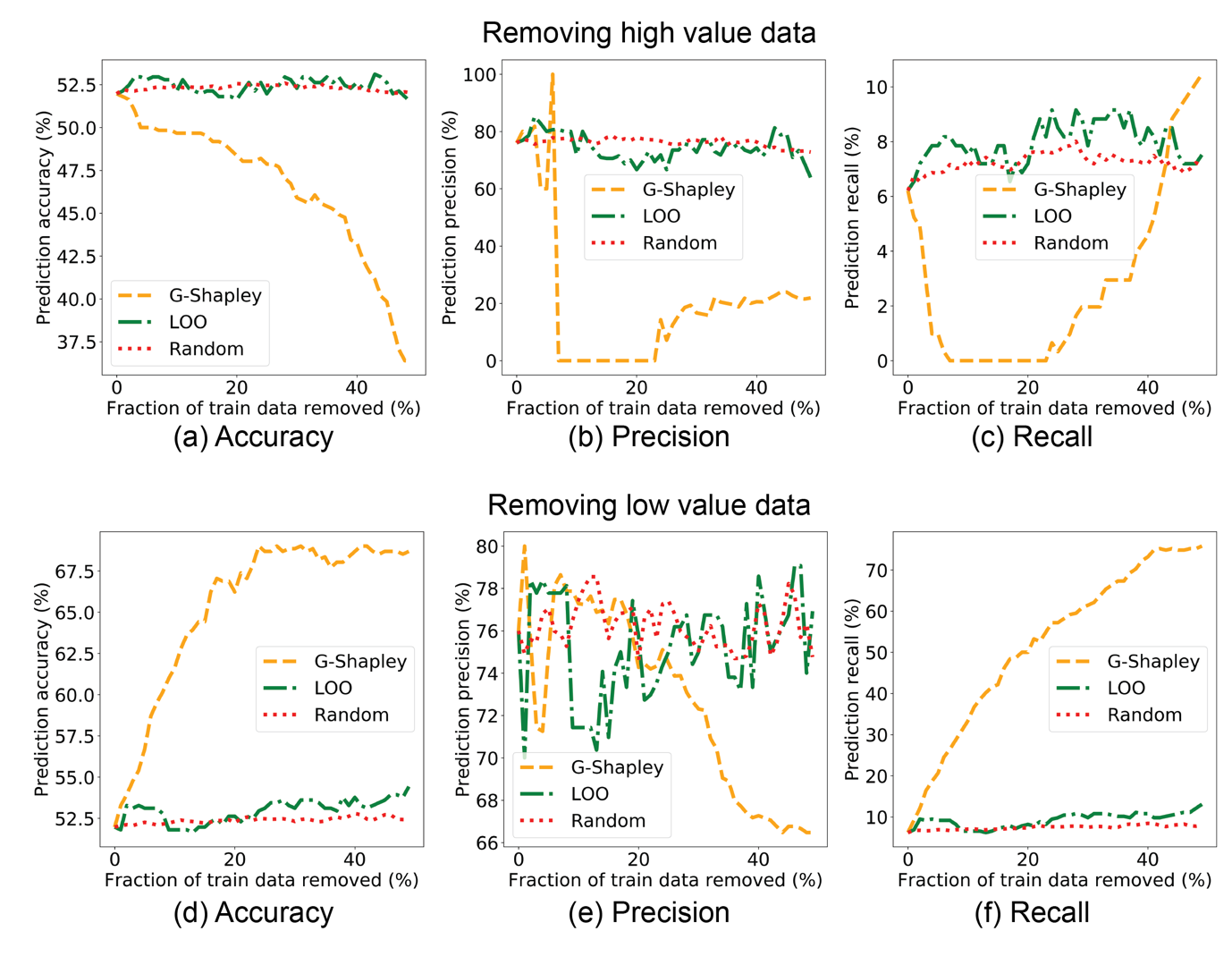
**

**Supplementary Figure S3.** In addition to TMC-Shapley approximation, we experimented with G-Shapley^3^ to approximate data Shapley values and obtained similar results as that with TMC-Shapley (Figure 2). **(a) - (c) Effects of removing high value data points to pneumonia detection performance**. We removed the most valuable data points from the training set, as ranked by G-Shapley, leave-one-out (LOO) and uniform sampling (random) methods. We trained a new logistic regression model every time when 1% of the training data points were removed. The x-axis shows the percentage of training data removed, and the y-axis shows the model performance on the held-out test set in terms of (a) accuracy, (b) precision and (c) recall. Removing the most valuable data points identified using G-Shapley method decreased the model performance more than using LOO or randomly removing data. We note that after removing more than 20% of the training data, the precision and recall scores for G-Shapley values increased, which might be because the percentage of positive cases increased after 20% of the training data were removed (see Supplementary Figure S2a). **(d) - (f) Effects of removing low value data points to pneumonia detection performance.** Conversely, we removed the least valuable data points from the training set. Removing the least valuable data points identified by G-Shapley method greatly improved the model performance in terms of prediction (d) accuracy and (f) recall.


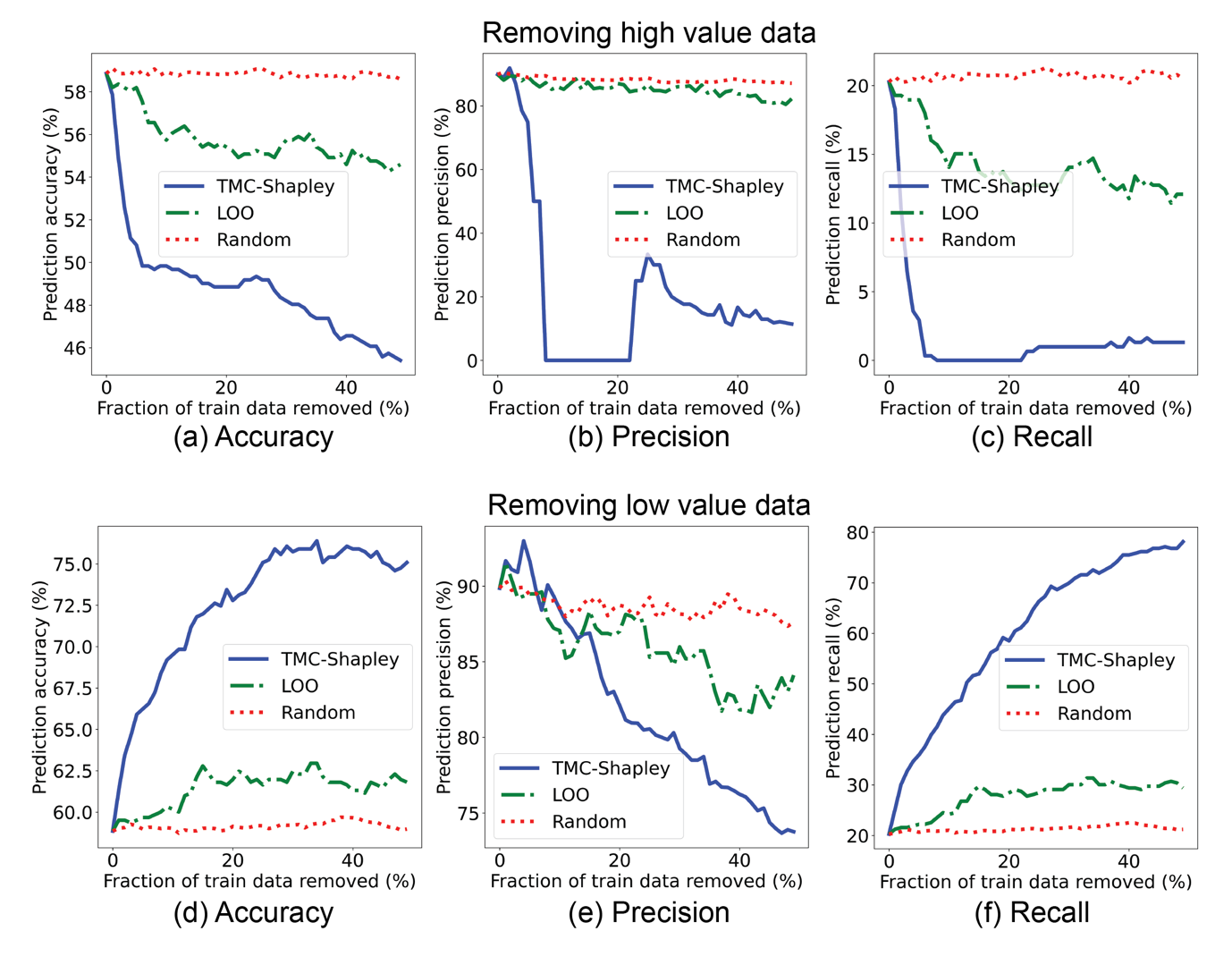


**Supplementary Figure S4.** We used features extracted from another pre-trained CNN, ResNet-50^2^, to compute TMC-Shapley values. We obtained similar results as that with pre-trained CheXNet^1^ (Figure 2). **(a) - (c) Effects of removing high value data points to pneumonia detection performance.** We removed the most valuable data points from the training set, as ranked by TMC-Shapley, leave-one-out (LOO) and uniform sampling (random) methods. We trained a new logistic regression model every time when 1% of the training data points were removed. The x-axis shows the percentage of training data removed, and the y-axis shows the model performance on the held-out test set in terms of (a) accuracy, (b) precision and (c) recall. Removing the most valuable data points identified by TMC-Shapley method decreased the model performance more than using LOO or randomly removing data. We note that after removing more than 20% of the training data, the precision scores for TMC-Shapley values increased slightly, which might be because the percentage of positive cases increased slightly after 20% of the training data were removed (see Supplementary Figure S2b). **(d) - (f) Effects of removing low value data points to pneumonia detection performance.** Conversely, we removed the least valuable data points from the training set. Removing the least valuable data points identified by TMC-Shapley method improved the model performance in terms of prediction (d) accuracy and (f) recall.


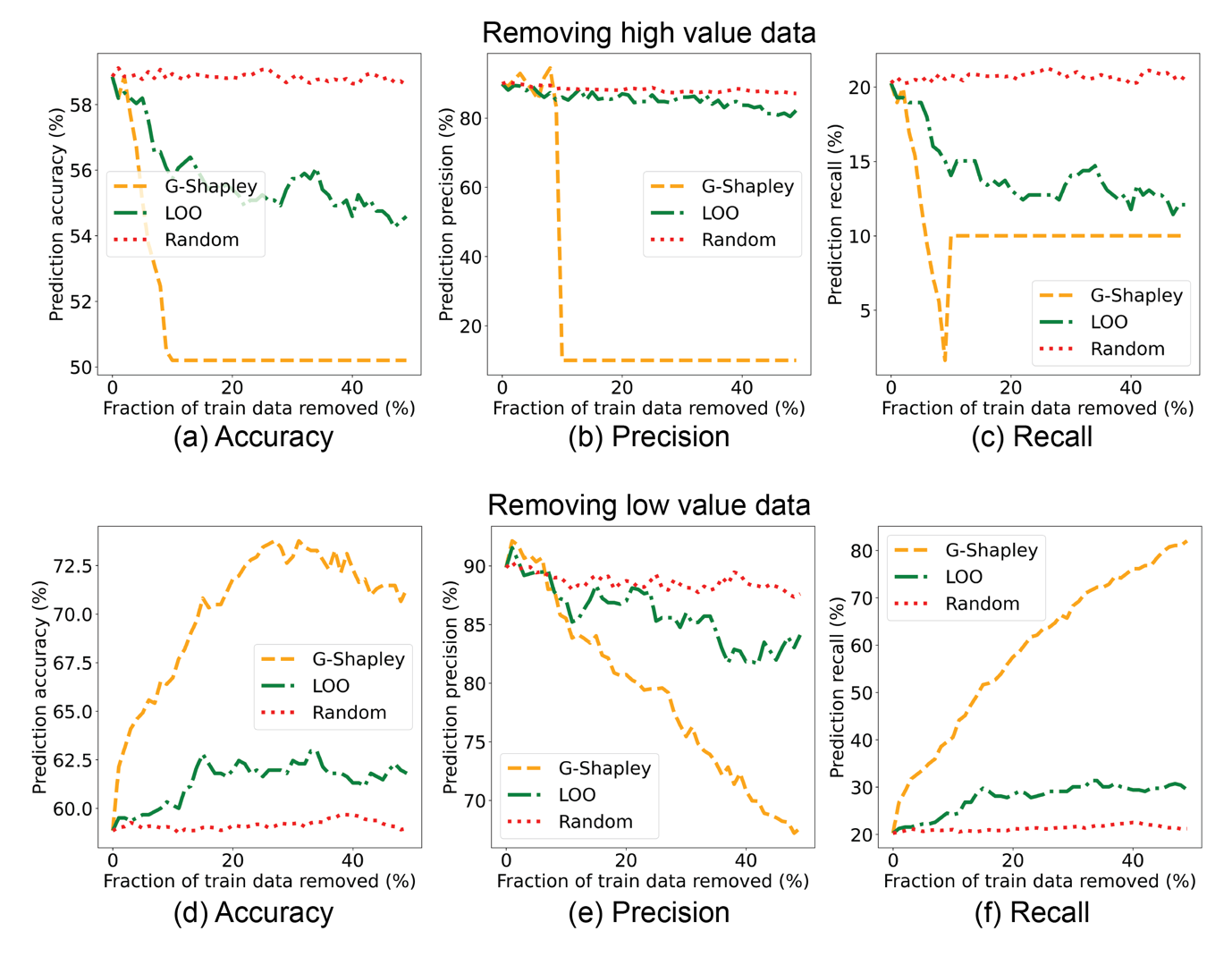


**Supplementary Figure S5.** We used features extracted from another pre-trained CNN, ResNet-50^2^, to compute G-Shapley values. We obtained similar results as that with pre-trained CheXNet^1^ (Supplementary Figure S3). **(a) - (c) Effects of removing high value data points to pneumonia detection performance**. We removed the most valuable data points from the training set, as ranked by G-Shapley, leave-one-out (LOO) and uniform sampling (random) methods. We trained a new logistic regression model every time when 1% of the training data points were removed. The x-axis shows the percentage of training data removed, and the y-axis shows the model performance on the held-out test set in terms of (a) accuracy, (b) precision and (c) recall. Removing the most valuable data points identified using G-Shapley method decreased the model performance more than using LOO or randomly removing data. **(d) - (f) Effects of removing low value data points to pneumonia detection performance.** Conversely, we removed the least valuable data points from the training set. Removing the least valuable data points identified by G-Shapley method greatly improved the model performance in terms of prediction (d) accuracy and (f) recall.


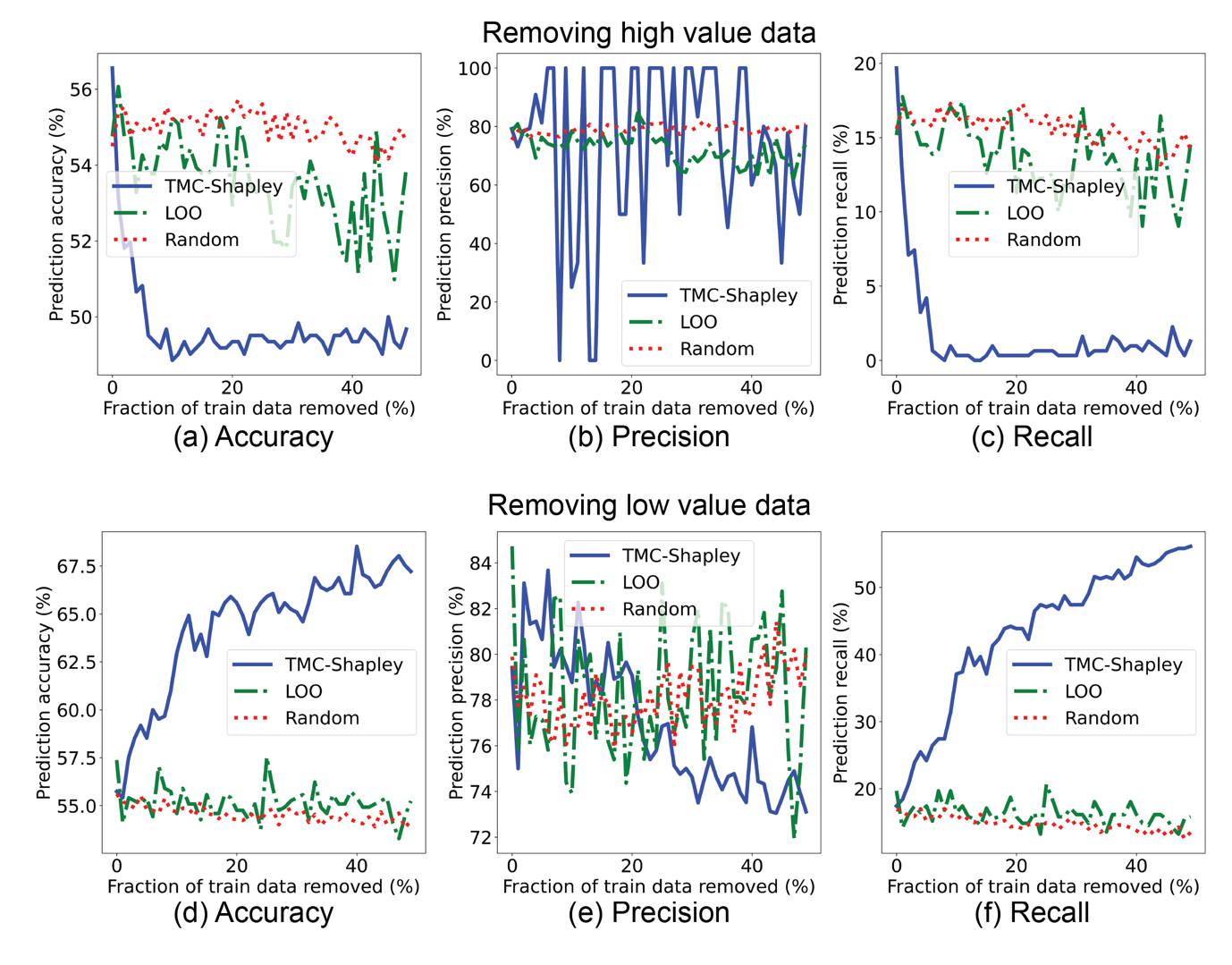


**Supplementary Figure S6.** We used the same features extracted from CheXNet^1^, but a different learning algorithm, multi-layer perceptron (MLP), to compute TMC-Shapley values. We obtained similar results as that with logistic regression (Figure 2). **(a) - (c) Effects of removing high value data points to pneumonia detection performance.** We removed the most valuable data points from the training set, as ranked by TMC-Shapley, leave-one-out (LOO) and uniform sampling (random) methods. We trained a new MLP model every time when 1% of the training data points were removed. The x-axis shows the percentage of training data removed, and the y-axis shows the model performance on the held-out test set in terms of (a) accuracy, (b) precision and (c) recall. Removing the most valuable data points identified by TMC-Shapley method decreased the model accuracy and recall more than using LOO or randomly removing data. **(d) - (f) Effects of removing low value data points to pneumonia detection performance.** Conversely, we removed the least valuable data points from the training set. Removing the least valuable data points identified by TMC-Shapley method greatly improved the model performance in terms of prediction (d) accuracy and (f) recall.


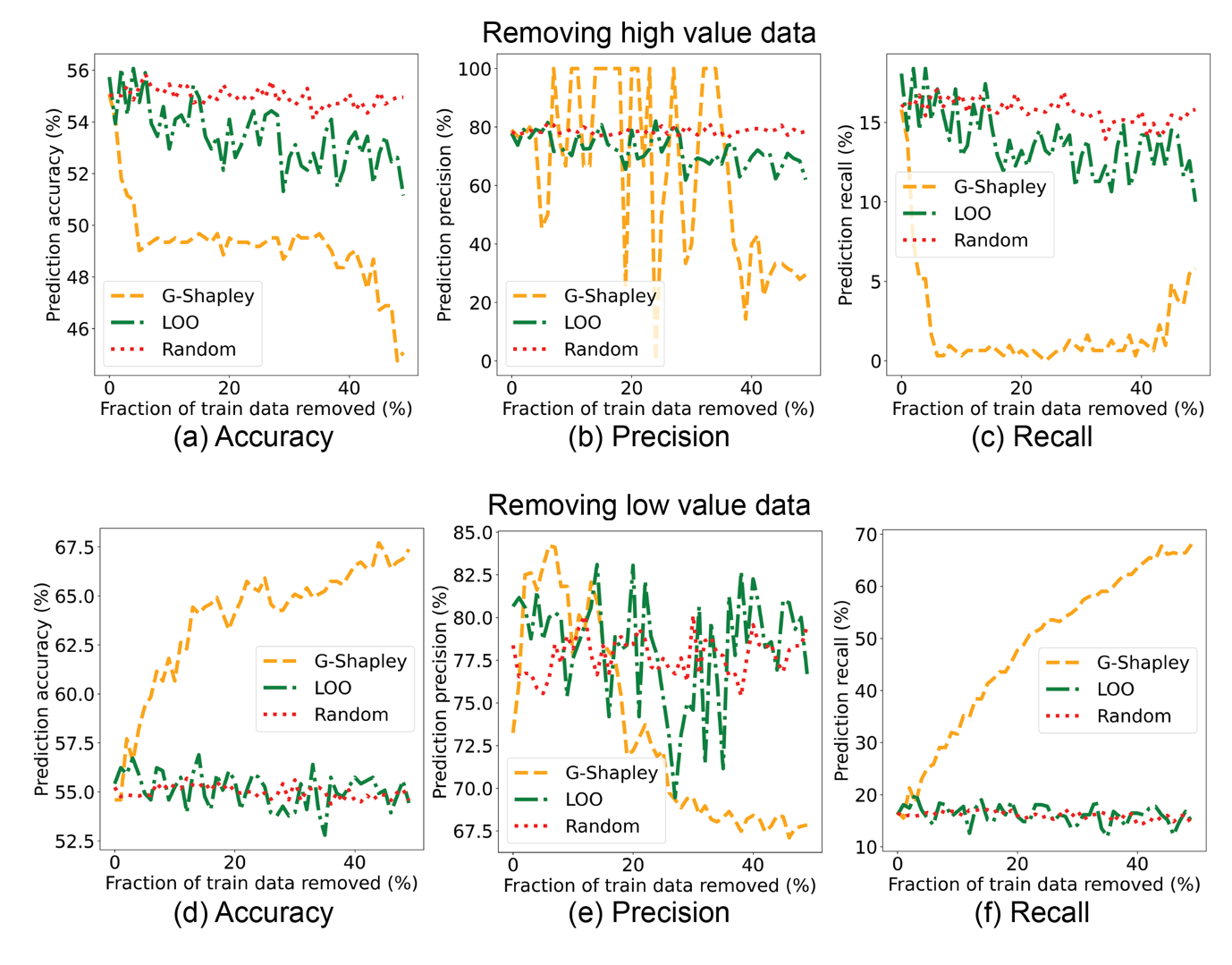


**Supplementary Figure S7.** We used the same features extracted from CheXNet^1^, but a different learning algorithm, multi-layer perceptron (MLP), to compute G-Shapley values. We obtained similar results as that with logistic regression (Supplementary Figure S3). **(a) - (c) Effects of removing high value data points to pneumonia detection performance**. We removed the most valuable data points from the training set, as ranked by G-Shapley, leave-one-out (LOO) and uniform sampling (random) methods. We trained a new MLP model every time when 1% of the training data points were removed. The x-axis shows the percentage of training data removed, and the y-axis shows the model performance on the held-out test set in terms of (a) accuracy, (b) precision and (c) recall. Removing the most valuable data points identified using G-Shapley method decreased the model accuracy and recall more than using LOO or randomly removing data. **(d) - (f) Effects of removing low value data points to pneumonia detection performance.** Conversely, we removed the least valuable data points from the training set. Removing the least valuable data points identified by G-Shapley method greatly improved the model performance in terms of prediction (d) accuracy and (f) recall.


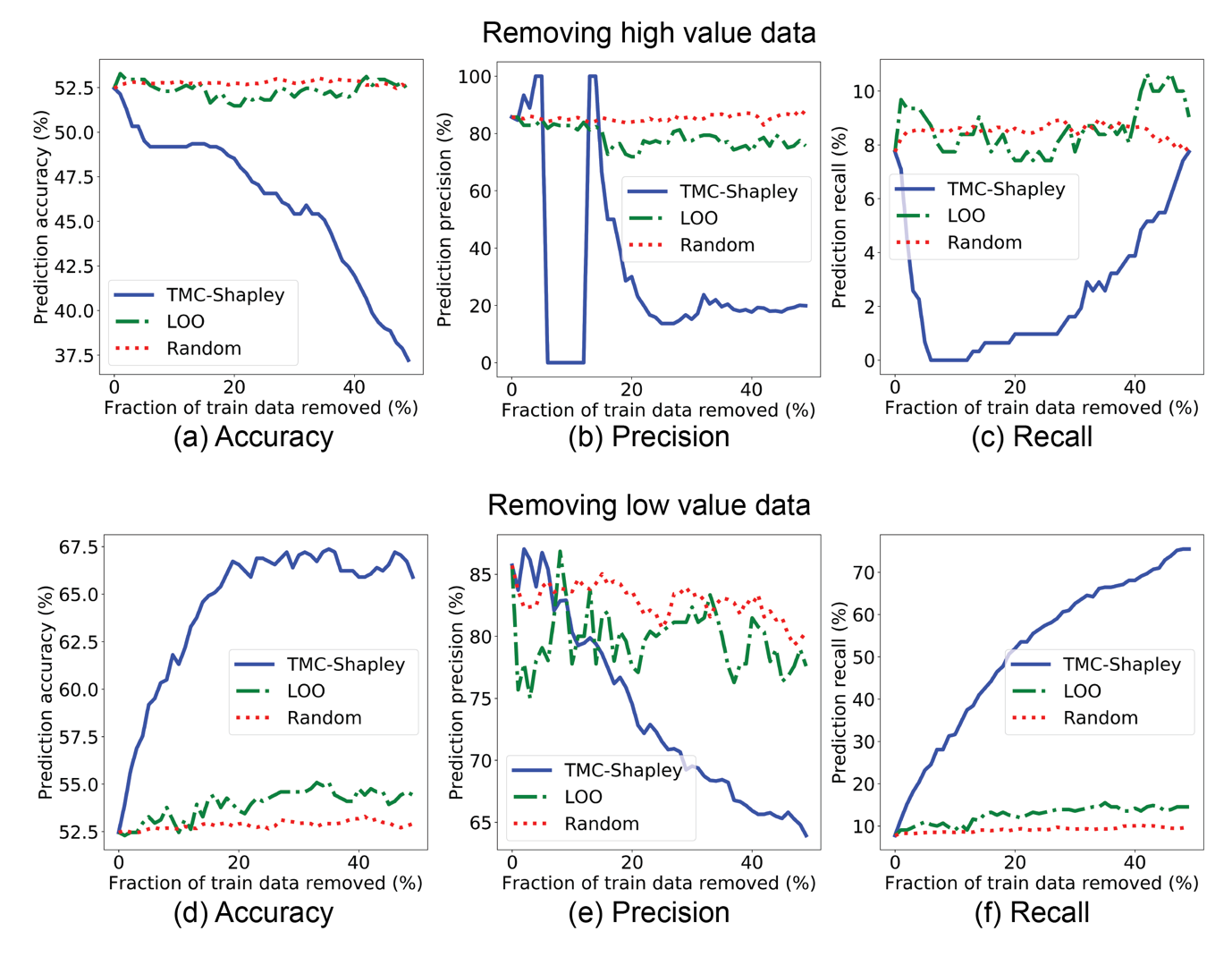


**Supplementary Figure S8.** We sampled different validation and held-out test sets to compute TMC-Shapley values for the training data. We obtained similar results as the original validation and held-out test sets (Figure 2). **(a) - (c) Effects of removing high value data points to pneumonia detection performance.** We removed the most valuable data points from the training set, as ranked by TMC-Shapley, leave-one-out (LOO) and uniform sampling (random) methods. We trained a new logistic regression model every time when 1% of the training data points were removed. The x-axis shows the percentage of training data removed, and the y-axis shows the model performance on the held-out test set in terms of (a) accuracy, (b) precision and (c) recall. Removing the most valuable data points identified by TMC-Shapley method decreased the model performance more than using LOO or randomly removing data. We note that after removing more than 15% of the training data, the precision and recall scores for TMC-Shapley values increased, which might be because the percentage of positive cases increased after 15% of the training data were removed (see Supplementary Figure S2d). **(d) - (f) Effects of removing low value data points to pneumonia detection performance.** Conversely, we removed the least valuable data points from the training set. Removing the least valuable data points identified by TMC-Shapley method greatly improved the model performance in terms of prediction (d) accuracy and (f) recall.


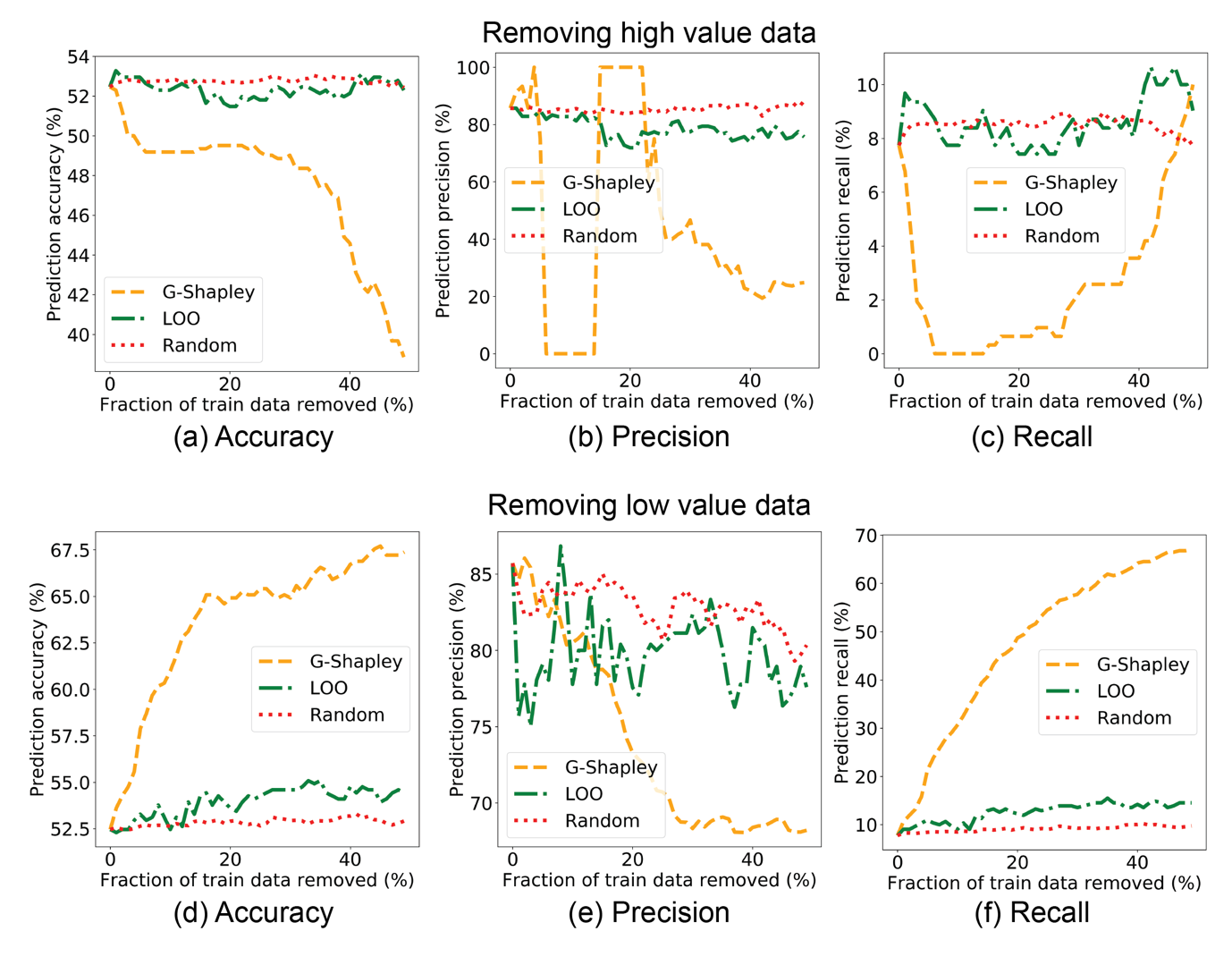


**Supplementary Figure S9.** We sampled different validation and held-out test sets to compute G-Shapley values for the training data. We obtained similar results as the original validation and held-out test sets (Supplementary Figure S3). **(a) - (c) Effects of removing high value data points to pneumonia detection performance**. We removed the most valuable data points from the training set, as ranked by G-Shapley, leave-one-out (LOO) and uniform sampling (random) methods. We trained a new logistic regression model every time when 1% of the training data points were removed. The x-axis shows the percentage of training data removed, and the y-axis shows the model performance on the held-out test set in terms of (a) accuracy, (b) precision and (c) recall. Removing the most valuable data points identified using G-Shapley method decreased the model performance more than using LOO or randomly removing data. We note that after removing more than 15% of the training data, the precision and recall scores for G-Shapley values increased, which might be because the percentage of positive cases increased after 15% of the training data were removed (see Supplementary Figure S2d). **(d) - (f) Effects of removing low value data points to pneumonia detection performance.** Conversely, we removed the least valuable data points from the training set. Removing the least valuable data points identified by G-Shapley method greatly improved the model performance in terms of prediction (d) accuracy and (f) recall.


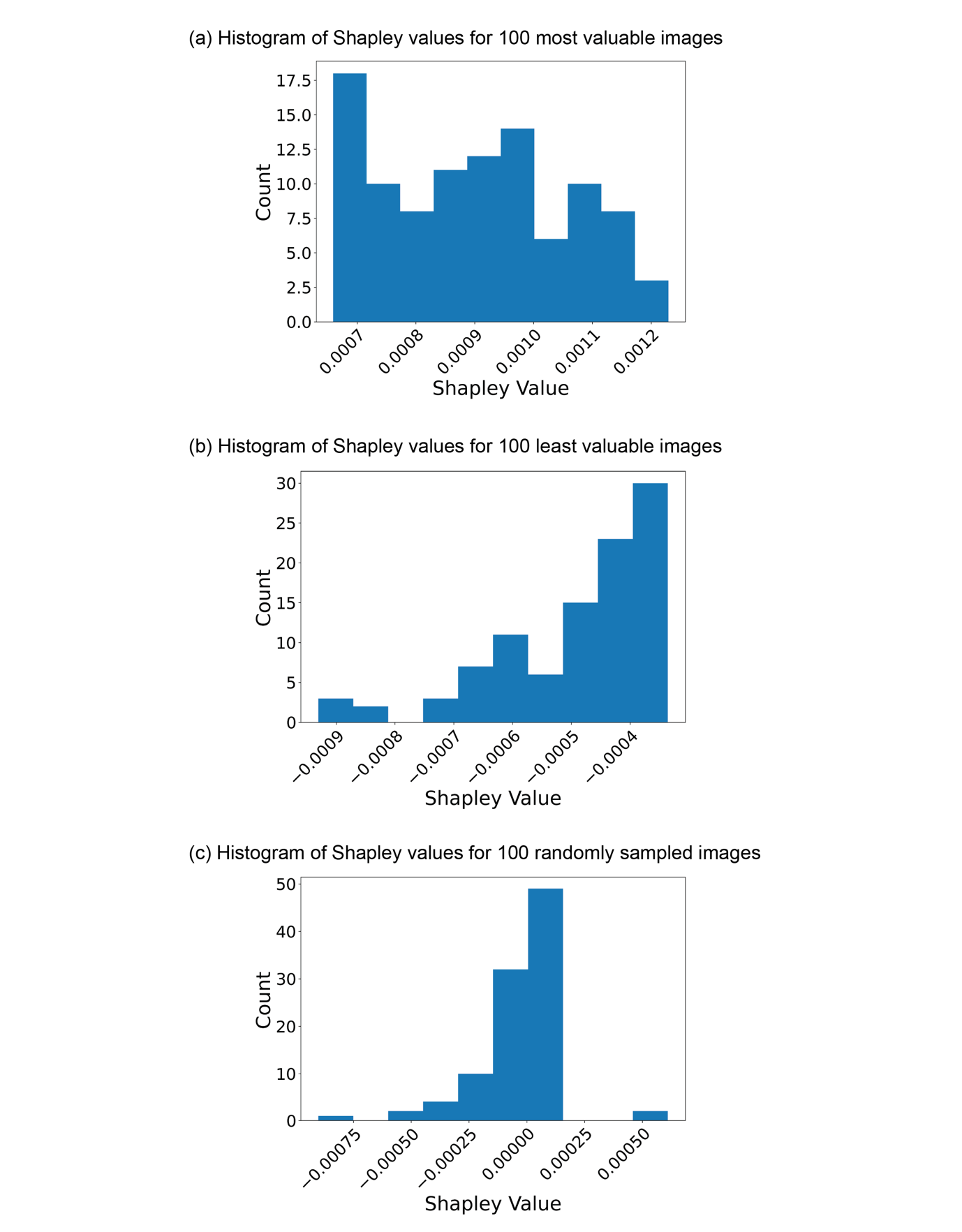


**Supplementary Figure S10. Histogram of TMC-Shapley values for** (a) 100 most valuable images, (b) 100 least valuable images and (c) 100 randomly sampled images in the training set.


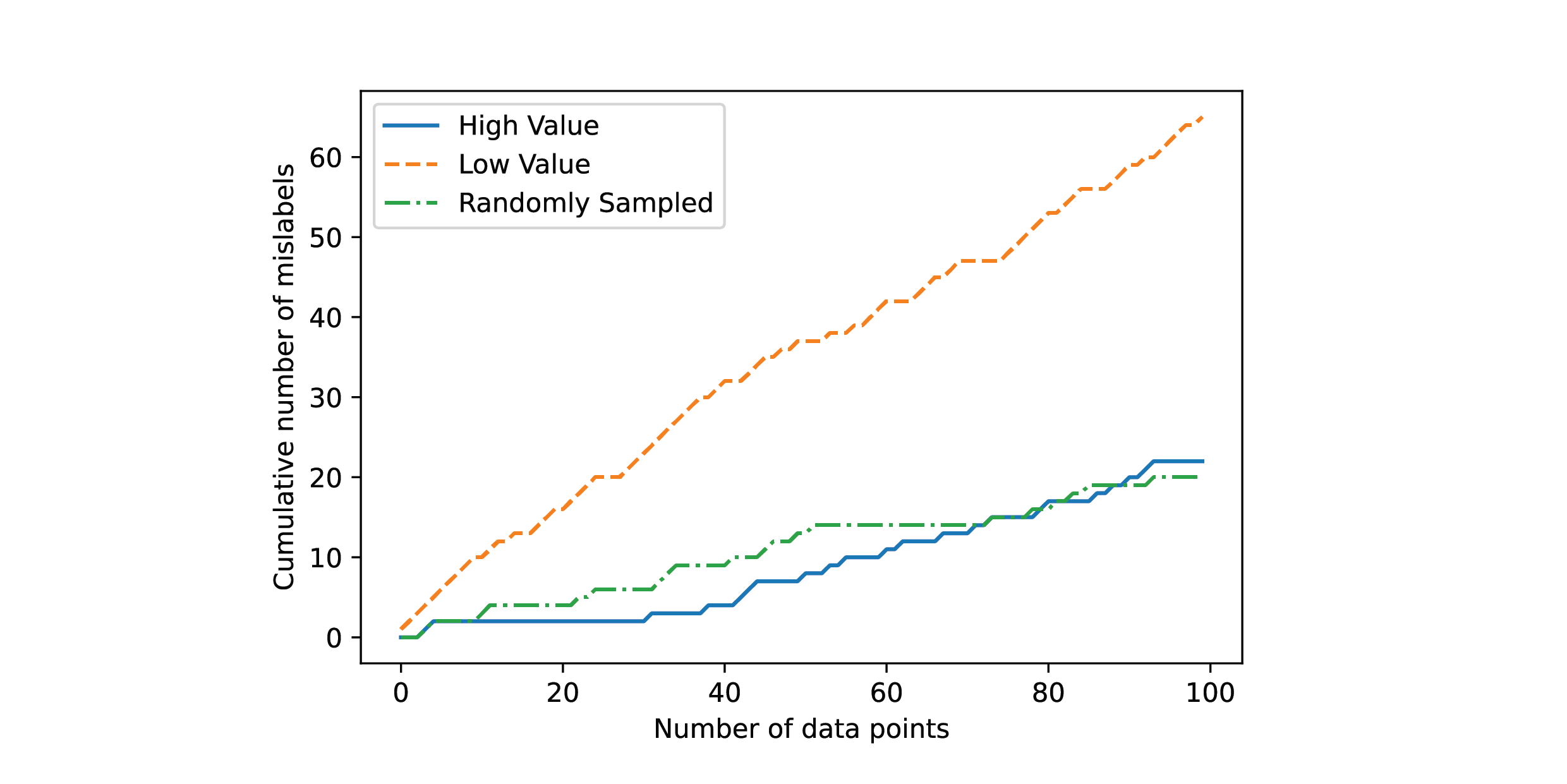


**Supplementary Figure S11. Cumulative number of mislabels in the 100 most valuable, 100 least valuable and 100 randomly sampled images in the training set.** The x-axis shows the number of data points visited. The y-axis shows the cumulative number of mislabels. For the 100 most valuable images (blue curve), the data points are sorted from the highest to the lowest Shapley value. For the 100 least valuable images (orange curve), the data points are sorted from the lowest to the highest Shapley value. For the 100 randomly sampled images (green curve), the data points are sorted randomly. Low value images had much more mislabels and a much steeper slope of accumulated mislabels compared to high value or randomly sampled images.


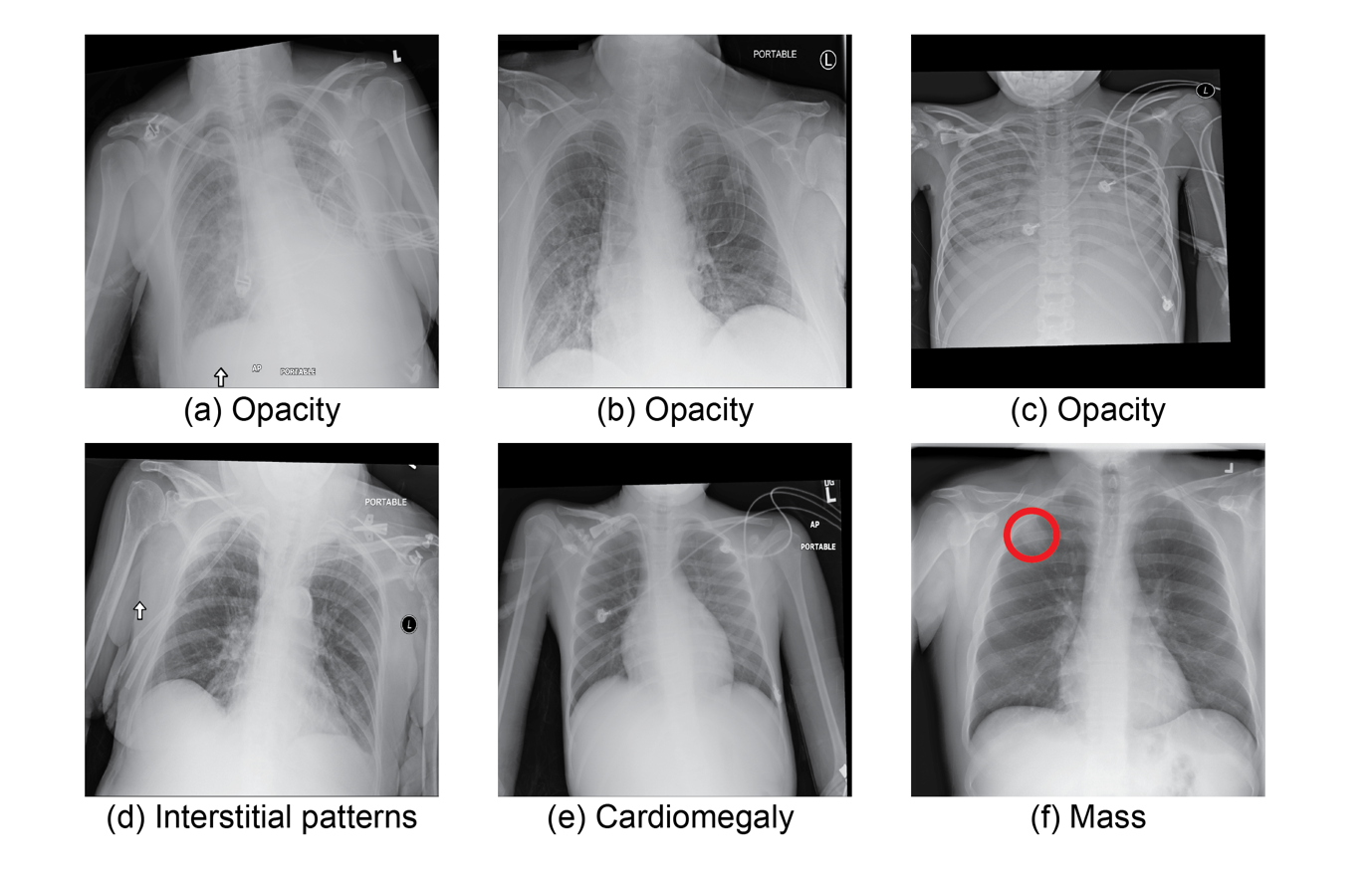


**Supplementary Figure S12. Example abnormalities observed in the mislabeled images.** (a)-(c) Bilateral opacity in both lung fields. (d) Abnormal interstitial patterns that are prominent in the upper lung areas. (e) Cardiomegaly (i.e. abnormally enlarged heart). (f) Abnormal mass in the upper right lung (circled in red).


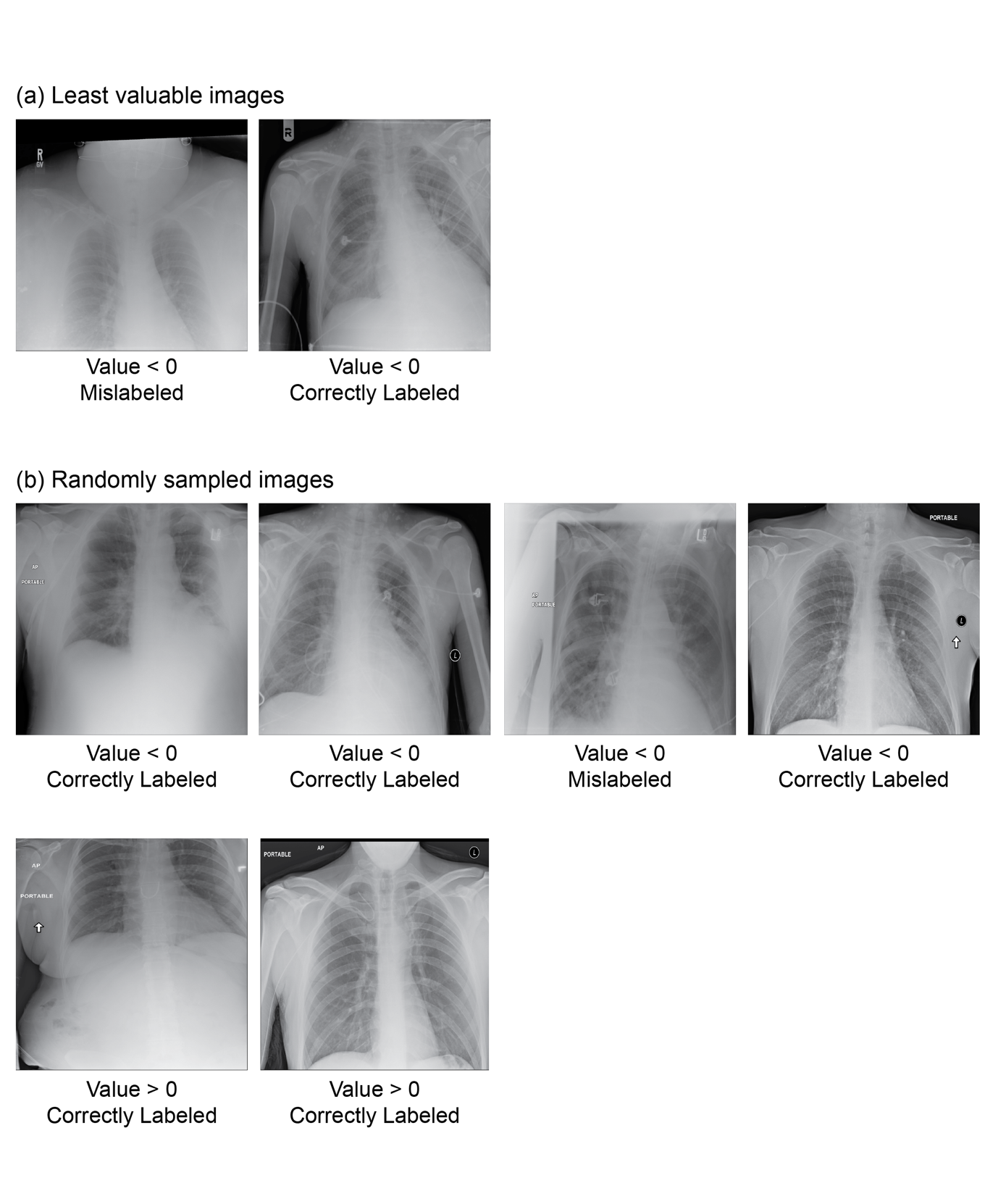


**Supplementary Figure S13. In the 300 chest X-ray images relabeled by the radiologists, there were eight images where a portion of the lung field was out of the image frame.** Six images had negative Shapley values whether or not they were correctly or incorrectly labeled, and the other two images had positive Shapley values and were correctly labeled. This suggests that low Shapley values not only indicate mislabels, but also poor image quality.

**References**

1. Rajpurkar, P. *et al.* Chexnet: Radiologist-level pneumonia detection on chest x-rays with deep learning. *arXiv Prepr. arXiv1711.05225* (2017).

2. He, K., Zhang, X., Ren, S. & Sun, J. Deep residual learning for image recognition. in *Proceedings of the IEEE conference on computer vision and pattern recognition* 770–778 (2016).

3. Ghorbani, A. & Zou, J. Data Shapley: Equitable Valuation of Data for Machine Learning. in *International Conference on Machine Learning* 2242–2251 (2019).
